# Casein-Based Biomaterials: Fabrication and Wound Healing Applications

**DOI:** 10.3390/molecules30153278

**Published:** 2025-08-05

**Authors:** Nikolay Estiven Gomez Mesa, Krasimir Vasilev, Youhong Tang

**Affiliations:** 1 Medical Device Research Institute, College of Science and Engineering, Flinders University, Adelaide, SA 5042, Australia; gome0105@flinders.edu.au; 2 Biomedical Nanoengineering Laboratory, College of Medicine and Public Health, Flinders University, Bedford Park, Adelaide, SA 5042, Australia; krasimir.vasilev@flinders.edu.au

**Keywords:** casein, micelles, wound healing, biomaterials, hydrogels, electrospinning, antibacterial, biocompatibility

## Abstract

Casein, the main phosphoprotein in milk, has a multifaceted molecular structure and unique physicochemical properties that make it a viable candidate for biomedical use, particularly in wound healing. This review presents a concise analysis of casein’s structural composition that comprises its hydrophobic and hydrophilic nature, calcium phosphate nanocluster structure, and its response to different pH, temperature, and ionic conditions. These characteristics have direct implications for its colloidal stability, including features such as gelation, swelling capacity, and usability as a biomaterial in tissue engineering. This review also discusses industrial derivatives and recent advances in casein biomaterials based on different fabrication types such as hydrogels, electrospun fibres, films, and advanced systems. Furthermore, casein dressings’ functional and biological attributes have shown remarkable exudate absorption, retention of moisture, biocompatibility, and antimicrobial and anti-inflammatory activity in both in vivo and in vitro studies. The gathered evidence highlights casein’s versatile bioactivity and dynamic molecular properties, positioning it as a promising platform to address advanced wound dressing challenges.

## 1. Casein: A Milk-Derived Protein

**Figure 1:**
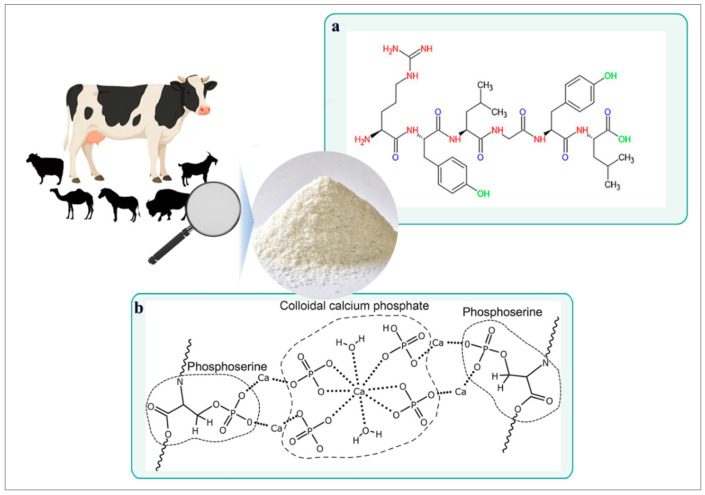
Chemical structure and sources of casein. (**a**) Representative peptide fragment of casein showing chemical structure and key functional groups [2]. (**b**) Schematic structure of a casein molecule highlighting phosphoserine residues and calcium phosphate bridges as pillars of stabilization [8].

Caseins belong to an extensive family of phosphorylated proteins, containing phosphate groups covalently bonded to amino acid residues—primarily serine [1]. These amino acids conform to the structure of casein, which carries chemically reactive functional groups, including phenolic hydroxyls, amino, hydrazine, and ketone groups [2], all interconnected via peptide bonds distinguished in polypeptides (Figure 1a). One of the primary industrial sources of casein is bovine milk; however, it is also found in other mammals, including goats, sheep, horses, buffalo, and camels [3,4]. Each of these contributes a variety of casein proportions, isoform profiles, and translational modifications [5].

Essentially, casein represents the principal protein fraction in the milk of ruminants, comprising approximately 80% of the total protein content [6]. In contrast, human and equine milk are classified as whey-dominant, with casein accounting for a smaller proportion of total protein (around 50%) [7]. The molecular characteristics illustrated in Figure 1b depict the phosphorylation capacity of casein, which is essential for calcium binding interactions. These interactions contribute to its supramolecular assembly, stabilized by van der Waals forces [8] and calcium bridges that link phosphoserine residues to calcium phosphate nanoclusters.

Bovine milk proteins are generally categorised into two major groups: caseins and whey proteins. The casein fraction comprises four primary isoforms—αS1-casein, αS2-casein, β-casein, and κ-casein—present at approximate mass proportions of 40%, 10%, 35%, and 15%, respectively [9,10]. These proteins are encoded by a tightly linked gene cluster on chromosome 6 [9]. The remaining protein components are attributed to predominant whey proteins: α-lactalbumin and β-lactoglobulin. Existing as large colloidal particles, casein subunits self-assemble into micelles, which are structurally stabilised by calcium phosphate nanoclusters [11]. In this conformation, micelles are characterised by an amphiphilic nature [12], containing both hydrophobic and hydrophilic domains. These complex intermolecular interactions confer the protein with unique functional properties relevant to nutritional, industrial, pharmaceutical, and biomedical applications.

Given its multifunctional profile, physicochemical versatility, and increasing biomedical interest, casein has emerged as a promising material for use in wound repair technologies. To identify and synthesise the existing knowledge on this topic, a targeted literature search was conducted across Scopus, Web of Science, and PubMed that encompassed publications from 2015 to 2025. Keywords used included “casein,” “milk protein,” “caseinate,” “wound healing,” “skin repair,” “electrospinning,” “hydrogel,” and “biomaterials.” This review aims to provide a comprehensive synthesis of casein’s structural and functional properties, highlighting its therapeutic potential in bioactive scaffolding, regenerative mechanisms, and material design in wound healing.

## 2. Structural and Functional Properties of Casein

### 2.1. Casein Micelle Structure

**Figure 2:**
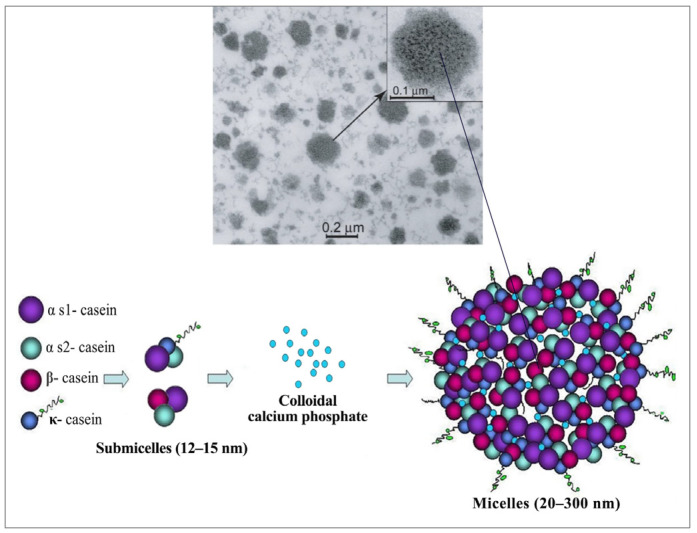
Transmission electron microscopy image (**top**) of a native casein micelle [16]. The lower panel illustrates the molecular structure of a bovine casein micelle, including submicelle composition and a proposed formation mechanism. Adapted from [15], with modifications in the lower panel based on [23].

The complex aggregates that form the internal structure of casein remain only partially understood despite extensive research [9]. The presence of numerous relatively hydrophobic regions in the interior has suggested that each phosphoprotein is adaptable, allowing it to acquire different conformations [13]. To address this challenge, some models have been proposed, with Walstra’s sub-micellar structure being the most widely accepted [1,14]. According to this design, micelles are constructed from uniform spherical subunits (12–15 nm) aggregated via hydrophobic interactions and calcium phosphate bridges [15]. In the interior, a hydrophobic core is assembled primarily from calcium-sensitive αS1, αS2, and β-caseins [16]. On the other hand, approximately 50% of κ-casein molecules are associated with glycosylation at their hydrophilic C-terminal domains [10]. This biochemical reaction enables κ-casein to form a stabilizing “hairy” layer [17] on the micelle surface (Figure 2), contributing to their notable polydispersity, net negative surface charge (~−20 mV), and strong water-binding capacity [10]. While this structural feature originates at the C-terminus, genetic modifications at the N-terminal—such as site-directed mutagenesis introducing glutamic acid or lysine residues—have been shown to modulate β-casein’s electrostatic interactions and self-association behaviour [18]. These transformations complement processing methods such as pH modification and high-pressure techniques, modulating a reversible micelle reformation and the encapsulation of bioactive in restructured casein micelles [19]. Moreover, enzymatic hydrolysis, ultrasonication treatments, and selective protein fractionation have been used to improve compound accessibility and interactions within the protein matrix [20]. The intrinsically disordered nature of caseins has enabled the characterization of distinct micellization and aggregation profiles between native and recombinant κ-casein, further underlining a fiber-forming potential [21]. These findings highlight the molecular adaptability of caseins. Natively proline-rich, they exhibit exceptional emulsifying capacity, surface activity, and structural flexibility [22]—features that enable the strategic engineering of tailored biofunctional platforms.

### 2.2. Casein-Based Biomaterials: Properties and Derivatives

Generally, casein’s structure has displayed versatile physicomechanical features, including a notable thermal stability [24]. Under certain heat treatments and varying pH conditions, the colloidal stability of casein micelles can be disrupted depending mostly on the C-terminal of κ-casein [25]. The heat-induced dissociation of this region or even its acidification [26] can disrupt the micellar integrity and protein unfolding, leading to visible flocculation and gelation [27]. These physicochemical transitions are fundamental to the formation of casein-based gels, which are characterised by significant absorption in aqueous environments. This describes a swelling property that is driven especially in hydrophilic polymer networks with a low crosslink density and high charge distribution [28], all typical features of phosphorylated casein chains. These water-binding properties are crucial to define casein’s functional characteristics, emphasizing its amphoteric versatility across various application domains.

Casein proteins are well-known for their pH sensitivity, becoming unstable below pH 5.0 [29]. This susceptibility forms the basis for their conventional extraction via isoelectric precipitation at pH 4.6 [30]. In addition to this approach, studies have explored enzymatic coagulation using rennet and membrane filtration techniques for casein isolation [15,31]. Casein’s commercial potential is frequently hindered by challenges in extraction efficiency and the stability of isolation methods. Recent advances in emerging techniques—such as ultrahigh-pressure homogenization, fluidized bed processing, and high-pressure homogenization—have demonstrated improved micelle disruption and enhanced β-casein recovery while preserving the structural integrity of milk’s bioactive constituents [32]. Ultrahigh-pressure homogenization, in particular, has been reported as a promising technique to modulate protein–polysaccharide interactions and micellar dissociation [33], thereby improving solubility and stability within aqueous biopolymer systems [34]. This is especially relevant given casein’s inherent insolubility in both water and organic solvents [35]. Furthermore, the acidic nature of casein, attributed to its free carboxyl and phosphate-derived hydroxyl groups, facilitates robust interactions with polyvalent metal ions such as Ca^2+^, Na^+^, K^+^, Zn^2+^, and Mg^2+^ [36]. These interactions lead to the formation of random coil caseinates, a family of milk protein derivatives that form stable micelles and exhibit high solubility [37,38].

In the biomaterials field, casein has demonstrated a rich profile that lends itself to diverse biomedical research [39]. Its unique mechanical properties have given it the capability for emulsion stabilisation [40] and even to form films [41]. Moreover, its high flexibility has allowed a drug carrier potential for microsphere-shaped casein in drug delivery [42]. Although casein and its salts are not currently listed in the FDA Inactive Ingredient Database, sodium caseinate has been nominated as a bulk drug substance under Section 503A of the U.S. Federal Food, Drug, and Cosmetic Act [43]. Its designation in Category 3 reflects a lack of adequate supporting data; however, this recent nomination underscores increasing pharmaceutical interest in casein-derived biomaterials. Characteristics such as bio-integration, high adaptability, low cost, and its secondary effects minimisation [44] position casein as a promising candidate for tissue engineering applications. In this context, the ideal biomaterial should have adequate mechanical strength as well as structural integrity to promote a favourable biomimetic environment for wound healing.

## 3. Wound Healing and the Role of Casein-Based Biomaterials

**Figure 3:**
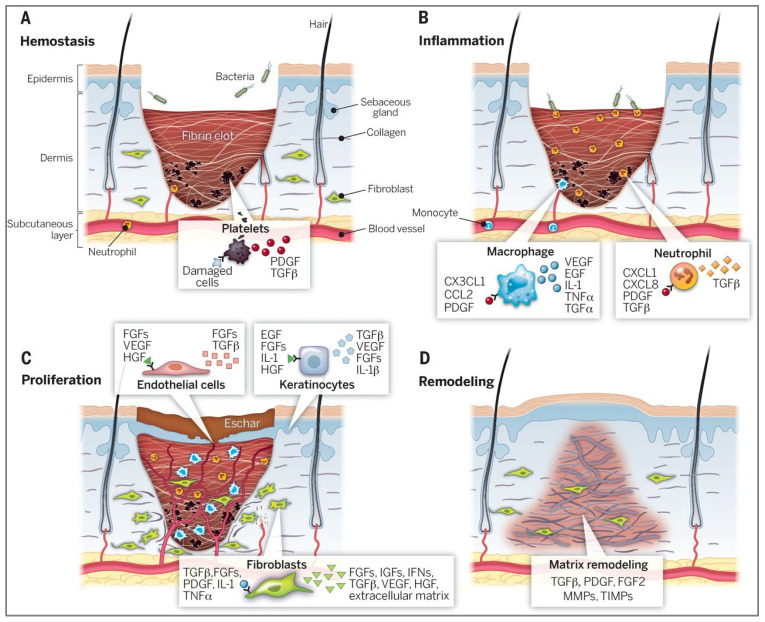
Four stages involved in wound healing process. (**A**) Hemostasis: Formation of a fibrin clot and activation of platelets stop bleeding and initiate the repair process through growth factor release such as TGF-β and PDGF. (**B**) Inflammation: Neutrophils and macrophages eliminate pathogens and secrete pro-inflammatory mediators. (**C**) Proliferation: Fibroblasts and endothelial cells drive extracellular matrix (ECM) formation, angiogenesis, and re-epithelialization. (**D**) Remodelling: Collagen fibers reorganize, restoring tensile strength and leading to scar tissue formation. The following abbreviations depict a subset of key factors illustrated. ECM, extracellular matrix; FGFs, fibroblast growth factors; HGF, hepatocyte growth factor; IFN, interferon; IGF, insulin-like growth factor; IL-1, interleukin-1; KGF, keratinocyte growth factor; MMP, matrix metalloproteinase; PDGF, platelet-derived growth factor; TGF, transforming growth factor; TIMP, tissue inhibitor of metalloproteinase; TNF, tumor necrosis factor; VEGF, vascular endothelial growth factor. Reproduced with permission from [47].

The skin acts as the body’s primary barrier, offering both physical protection and immune defence against external threats [45]. When this is disrupted, it initiates a complex wound healing response involving biological stages that restore tissue structure and function [46]. The process is divided into four main phases: haemostasis, inflammation, proliferation, and remodelling (Figure 3). Immediately following a tissue injury, haemostasis occurs through vasoconstriction and platelet aggregation, forming a fibrin clot that provides an initial barrier and prevents excessive blood loss [47]. The release of growth factors such as factor-beta (TGF-β) and platelet-derived growth factor (PDGF) initiates the respective healing process. The following inflammatory phase is marked by neutrophils, monocytes, and white blood cells, facilitating the eradication of pathogens and damaged cells [47]. During this phase, these key cells release soluble mediators such as proinflammatory cytokines (including IL-1 and TNF-α) and growth factors involved in the activation of epithelial cells that promote inflammation and prepare the wound for healing [48]. The proliferative phase is not limited to a certain timeframe, occurring continuously in the background [49]. Fibroblasts start migrating to the wound site and actively create extracellular matrix (ECM) components, which further enhance cellular migration [50]. As healing advances to the final remodelling stage, the collagen fibres already generated become more structured, resulting in the restoration of tissue strength and scar formation [51].

Particularly, wound management represents a critical challenge due to its rising prevalence and infection management-associated costs, estimated to cost up to 96.8 billion in healthcare spends annually [52]. Although existing therapeutic strategies such as bioengineered skin substitute applications have made promising clinical progress, these treatments are not yet adequate to fully restore anatomical and physiological skin integrity [53]. Persistent infection and excessive exudate production significantly hinder the effective management of chronic wounds [54]. These challenges cause exudate accumulation, leading to tissue maceration [55], impeded cellular migration, and prolonged healing [54]. Additionally, infections are often driven by inflammatory responses and biofilm formation [49], which impact the recovery process and elevate systemic health risks.

To tackle these interconnected healing challenges, recent studies have focused on nature-derived biomaterials possessing an inherent composition of bioactive molecules and controllable nanostructures [56]. Milk-based proteins, in particular, offer promising solutions due to their structural versatility, antioxidant potential, and ability to support tissue regeneration [57]. Proteins and peptides from fermented dairy products, cow milk, camel milk, and even soybean milk have shown notable efficacy in accelerating wound closure [58,59], mitigating oxidative stress [60], and enhancing angiogenesis [61]. Furthermore, milk-derived extracellular vesicles and exosomes have been identified as powerful modulators of inflammation cytokines [62], promoting collagen synthesis [63] and the epithelial repair of wound tissues [64,65]. The emergence of colostrum-derived exosomes has revealed a rich protein profile—including casein, β-lactoglobulin, α-lactalbumin, and lactoferrin [66]—capable of modulating immune regulatory responses [67,68] and promoting cellular proliferation [69,70]. Among these macromolecules, β-casein has been shown to activate kinase pathways associated with cellular growth [71], thereby contributing to wound site regeneration. While various milk-derived modalities possess therapeutic value, casein molecules have gained particular attention as a bio-functional material for engineering skin-regenerative systems.

Specifically, casein offers numerous advantageous properties as a biomaterial, including biocompatibility, intrinsic bioactivity, and low immunogenicity [72]. The high content of amino groups (−NH_2_) in casein allows surface functionalisation, resulting in the promotion of cellular adhesion [73]. Their high water content and swelling capabilities have allowed the effective absorption of excess exudate [74], maintaining optimal wound conditions. In fact, a moist environment is conducive towards epithelialisation, which facilitates cellular migration [75], enabling effective wound coverage and the restoration of the skin’s protective function [76]. Additionally, casein exhibits inherent antioxidant properties [77], which can decrease microbial burden and oxidative stress at the wound site. When engineered as drug carriers for antiseptic agents like polyhexanide, casein-based materials have demonstrated potent activity against pathogens, further enhancing their therapeutic potential [74]. These functional advantages are reinforced by the intermolecular interactions within casein micelles, which support the fabrication of nanoparticles, fibres, membranes [78], and non-toxic hydrogels [74], expanding its range of application through diverse fabrication methods.

## 4. Fabrication of Casein-Based Wound Dressings

### 4.1. Casein-Based Hydrogels

Hydrogels possess a three-dimensional network structure and typically exhibit mechanical properties and high water content that mimic the characteristics of native skin [79]. Caseinates, capable of forming micellar structures, have attracted interest for encapsulating both hydrophilic and hydrophobic bioactive compounds. However, its low water vapor resistance and inherent fragility require it to be mixed with other materials [40]. Therefore, mostly in scaffold applications, caseinates have been employed in combination with complementary polymers to enhance mechanical stability and integrity [80].

Structurally, casein micelles facilitate gel formation via non-covalent interactions—including hydrophobic forces, hydrogen bonding, and electrostatic repulsion—between unfolded polypeptide regions, resulting in dynamic physically crosslinked networks [81]. Beyond these static interactions, casein’s aggregation behavior reflects a complex interplay of molecular conformation, interfacial adsorption, and rheological characteristics across multiple scales [82]. Its capacity to transition between colloidal micelles, partially denatured chains, and random-coil states under varying conditions allows for the adaptive structuring of hydrogel networks [83,84]. Casein has been utilized as a protein model within polyacrylamide hydrogels, generating highly stretchable and tough composites with a reported fracture stress of 180 kPa and strain exceeding 2000% [81]. Moreover, calcium ion release from casein hydrogels has been shown to activate calcium-binding proteins, modulate intracellular enzyme activity, and stimulate the synthesis of skin proteins and sebum, thereby accelerating epidermal repair [85,86]. These outcomes underscore the role of engineered casein–hydrogel systems in harnessing protein–matrix interactions to drive both mechanical tissue characterization and biological responsiveness [78]. Building on these principles, recent developments have explored casein-based hydrogels featuring enhanced bioactivity and more accessible synthesis routes.

**Figure 4:**
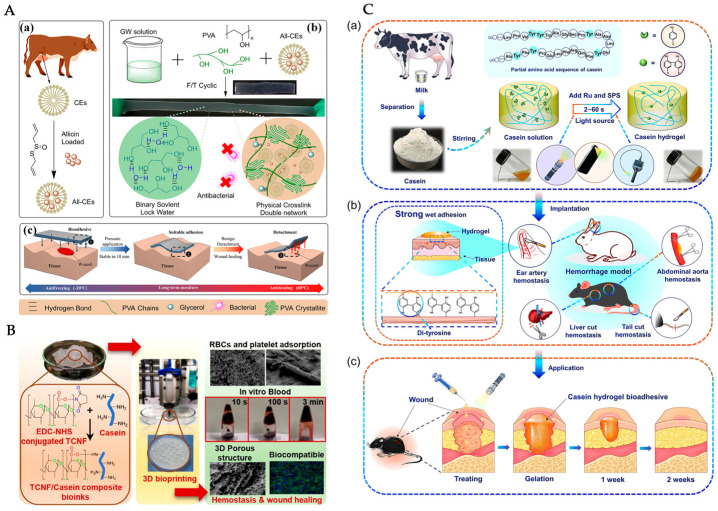
Representative casein-based hydrogel systems for wound healing. (**A**) Schematic of casein micelles (CEs) in an organohydrogel fabrication, illustrating (a) allicin load design, (b) synthesis, formation, and tensile mechanisms, and (c) adhesive tissue properties [87]. (**B**) Fabrication of cellulose nanofibrils (TCNFs)/casein composite bio-inks via 3D bioprinting, supporting blood cell adsorption, biocompatibility, and wound repair [88]. (**C**) White-light activated milk protein bioadhesive for rapid hemostasis and wound healing. (a) Casein mixed with tris dichlororuthenium (II) hexahydrate (Ru), and sodium persulfate (SPS), is exposed to visible light (2–60 s), initiating di-tyrosine crosslinking and hydrogel formation. (b) Covalent di-tyrosine bonding at the tissue interface enables robust wet adhesion and rapid hemostasis in various arterial and visceral haemorrhage models. (c) Application to skin wounds promotes closure and progressive tissue regeneration over one to two weeks in vivo [72].

For instance, an innovative casein micelle-based organohydrogel engineered by the simplicity of a one-pot synthesis was presented [87], involving the dissolution of a casein–allicin suspension. Hydrogel fabrication was achieved through a cyclic freezing–thawing procedure. The formulation was combined with polyvinyl alcohol (PVA) and glycerol, leading to a highly stretchable and moisture-retentive material. In Figure 4A, it can be seen that the multi-faceted interaction mechanisms enabled stable adhesion and structural integrity. In particular, their hydrogel demonstrated excellent adherence to both dry and wet tissue, which was attributed to hydrogen bonding and physical interactions between the hydrogel network and the tissue surface [87]. The organohydrogel also retained high swelling capacity and promoted cell proliferation and antimicrobial properties against bacteria. The versatility of casein as a bifunctional element in the hydrogel fabrication is shown in Table 1.

**Table 1:** Overview of recent studies using casein as a bio-functional component in hydrogel systems.

Hydrogel Composition	Fabrication Method	Key Outcomes and Wound Healing Relevance	Ref.
Casein micelles, PVA, glycerol–water solvent	Organohydrogel formation via self-assembly	Mechanical strength, tissue adhesion for wound healing, moisture retention, and antibacterial properties	[87]
Cellulose nanofibrils, chitosan, casein	3D bioprinting of composite bio-inks	Blood clotting acceleration and fibroblast proliferation; potential use in blood reduction for traumatic hemorrhage episodes	[88]
Casein, tris dichlororuthenium (II) hexahydrate (Ru), and sodium persulfate (SPS).	White-light-induced crosslinking using ruthenium-induced catalysis	Ultrafast gelation, bioadhesive, highly adaptable and suitable for first aid wound treatment	[72]
Sodium caseinate (Na Cas), gelatine, thyme oil	Essential oil encapsulation in Na Cas micelles via solvent evaporation method	Antibacterial activity through bacterial membrane disruption; in vivo wound healing potential, within ~15% faster epithelialization	[89]
Casein sodium salt, acid casein, Octiset^®^ or polyhexanide	Synthesis via acrylamide polymerization and inducing casein micelle coagulation	Sustained drug release and antimicrobial activity against common wound pathogen	[74]
Polyacrylamide, casein micelles		High elasticity and durability; mechanical resilience and notch-insensitive gels for tissue engineering	[90]

In another study, a casein-based bio-printed scaffold with the incorporation of cellulose nanofibril was synthetised [88] in a conjugate with eucalyptus kraft pulp. By using a 3D-bioprinter, their composite construct not only accelerated blood clotting but also fibroblast proliferation [88], which supports cell growth in wound healing stages. For emergency first-aid treatment, the synthesis of a cross-linkable milk protein hydrogel with ultrafast gelation properties was similarly reported [72]. By forming di-tyrosine bonds in the presence of a ruthenium-based catalyst (Ru) [72], the samples completed gelation when exposed to white light in a base solvent. As a result, a bioadhesive material showing strong tissue adhesion and rapid sealing capability was obtained for use in irregular wound shapes.

Other caseinate derivatives have been explored for the creation of nanocomposite hydrogels loaded with different agents. A sodium caseinate/gelatine hydrogel loaded with thyme essential oil [89] demonstrated robust antibacterial activity and enhanced in vivo wound healing with approximately 15% faster closure rates compared to controls. Also, the incorporation of antiseptic agents such as Octiset^®^ into casein-based hydrogels was explored [74]. These casein hydrogels were synthesised through the free radical polymerisation of acrylamide combined with micellar aggregation. Mainly, the formulations exhibited potent antimicrobial efficacy against *Staphylococcus aureus* and *Pseudomonas aeruginosa*, alongside key factors in chronic wound management such as biocompatibility and controlled drug release. Some hydrogel researches are centred on drug delivery, where highly stretchable materials [90] and casein scaffold nanocomposites have shown an effective release of various drugs [91,92] suggesting potential use for tissue engineering scenarios.

### 4.2. Films

Casein’s film-forming ability has also been explored for wound dressing applications. Alginate dialdehyde (AD)-crosslinked casein films were developed [93], highlighting their enhanced water absorption behaviour and improved structural integrity. The suitability of these films for high exudating wounds could be identified through moist permeation studies. Moreover, solvent casting techniques to fabricate functionalised casein films have been employed with specific wound dressing relevance [94,95]. A silver–casein composite film, where silver nanoparticles formed in situ within the polymer matrix, showed improvements in barrier properties and antibacterial performance [94]. Designed to respond to protease activity, a film containing fluorogenic peptide substrate that modulates excessive proteolysis in chronic wounds has also been reported [95]. As the film degraded, it reduced protease levels and liberated antioxidants, contributing to enhanced tissue recovery.

### 4.3. Fibers/Mats

**Figure 5:**
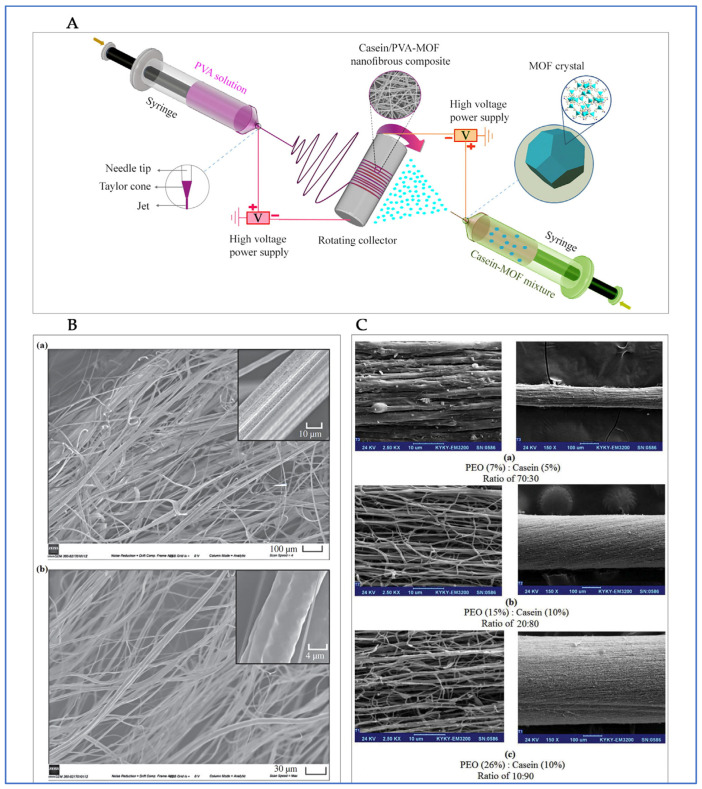
Overview of casein-based fibres for wound healing. (**A**) Schematic representation of a casein/PVA-MOF nanofiber composite fabrication with the incorporation of zinc-based metal–organic frameworks [110]. (**B**) Fibre surface of (a) PCL (6.2 ± 2.3 µm avg diameter) and (b) PCL/casein fibres (1.4 ± 0.5 µm avg diameter) via pressurized gyration, demonstrating uniform fibre distribution [98]. (**C**) Morphological comparison of nanofiber yarn PEO/casein produced at different concentrations and three blend ratios (70:30, 20:80 and 10:90), revealing different fibre arrangements [96].

Another extensive platform for wound healing is the development of casein-based fibres. Some examples of fabrication methods for the obtention of casein-derived fibrous materials include extrusion, chemical crosslinking, and electrospinning [96,97]. A pioneering study proved the wound healing potential of pure casein fibres produced through wet spinning [98]. For this procedure, fibres were obtained via pressured gyration mixing casein and polycaprolactone (PCL) (Figure 5B). These fibres demonstrated mechanical durability, swelling capacity, and in vivo wound closure efficacy comparable to commercial collagen dressings [98]. The study also underlined casein’s immunomodulatory capability and the formation of new blood vessels, emphasizing its potential as a therapeutic fibre matrix.

Among the various techniques, electrospinning stands out due to its ability to produce nanofibres with high porosity and surface area. Also, electrospun fibres have been recognized for mimicking the ECM, conducive to epithelial cell proliferation [99]. This method uses a high-voltage electric field to bring polymer solutions into fine fibres, which solidify on a determined collector as the solvent evaporates [40]. Since the non-spinnable capability of casein has been reported, the addition of a polymers is crucial for a supportive mechanical matrix [100]. PCL in combination with gelatine and casein to fabricate electrospun scaffolds was explored for tissue engineering applications [101]. The resulting nanofibrous material exhibited uniform, bead-free fibres with a mean diameter of approximately 259.27 ± 56.33 nm and a pore size of 15.98 ± 2.14 µm. Cellular responses and biological assessments confirmed the development of biomimetic tissues as a desirable candidate for cartilage regeneration. Mechanical characterisation further revealed that the inclusion of 2 wt% casein enhanced hydrophilicity and the tensile strength of the fibres to 9.8 ± 1.6 MPa [101].

Despite its intrinsic brittleness, pure casein fibre systems can be effectively modified through blending and crosslinking strategies [102]. Casein’s chemical compatibility with various biopolymers enables the development of composites with improved mechanical performance. For example, reinforcement with recycled cellulose fibres enhances both strength and flexibility [103], whereas casein mats containing 1 wt% κ-carrageenan exhibit more modest mechanical properties (tensile strength ~0.2 MPa; stiffness ~12 MPa) [104]. To address these limitations and promote elasticity and swelling, plasticizers such as glycerol have been employed to form porous 3D networks that enhance both durability and bifunctionality [105]. As a recent alternative, enzymatic crosslinking can further improve water-binding capacity and reduce brittleness in spinning micellar casein fibres [106,107]. Chemical crosslinkers can change casein protein environment interactions to influence specific fibre features. For instance, electrospun casein/PEO fibres crosslinked with TA produced stable, water-insoluble networks (~2 μm diameter), nearly doubling tensile strength (from 0.91 to 1.88 MPa) and tripling elongation at break (from ~94% to ~275%) [108]. Although chitosan/gelatine films similarly reinforced with tannic acid and nanocellulose may exhibit higher tensile strength [109], casein-based fibres offer exceptional elongation (~275%) and present a sustainable, bioactive textile solution derived from waste milk. This sustainable approach supports circular bioeconomy efforts and continues to gain interest through its compatibility with diverse materials that enhance electrospinning capability and expand biomedical applicability.

Polyvinyl alcohol (PVA) is one of the most commonly utilized co-polymers for electrospun caseinate derivatives. For instance, casein/PVA nanofibers with a zinc-based metal–organic framework (MOF) were developed [110]. By implementing double syringe pumps through electro-spraying and electrospinning techniques (Figure 5A), bead-free nanofibers with an average diameter between 80 and 150 nm were achieved. Research showed the casein/PVA improvement of the wettability performance and an advantageous porosity, as well as evidence of efficient activity against bacteria and haemostatic properties. Similarly, a study determined different Casein/PVA mass ratios for the obtention of nanofibers [111]. The optime uniform mat, evidencing reliable blood clot formation and haemostasis acceleration, was found at 40:60. Additionally, sodium caseinate (SC) has been explored with PVA, implementing a single-fluid electrospinning and selecting glutaraldehyde (GLA) as the cross-linking agent [112]. In terms of their mechanical performance, the resulting electrospun mats demonstrated a notable improvement, with the PVA/SC mixture increasing the breaking stress. Further enhancements were achieved through the incorporation of nanoparticles and other additives. Specifically, the addition of copper oxide (CuO) nanoparticles was shown to improve both the breaking strength and elongation behaviour of the mats [113], while the inclusion of reduced graphene oxide (rGO) led to an increase in tensile strength [114]. These findings indicate that the mechanical properties of PVA/SC electrospun mats can be effectively optimized by carefully selecting cross-linking agents and reinforcing fillers. Surprisingly, the matrix involving zinc oxide nanoparticles depicted high toxicity as reported in the cell viability findings. However, the PVA/SC nanofiber in situ method can be adopted to fabricate fibroblast tissue material [112].

Lastly, polyethylene oxide (PEO) has proven to be a valuable polymer in facilitating the electrospinning of milk protein. Casein was electrospun with PEO in the presence of silver nanoparticles [115], producing nanofibers with strong antibacterial activity against *Staphylococcus aureus* and *Escherichia coli*, highlighting a potential application for infection-prone wound sites. Likewise, casein/PEO nanofibrous yarns [96] with promising morphology and fibre uniformity (Figure 5C) were developed, indicating their suitability for biomedical textiles and wound coverage. A more recent study utilised casein sourced from revalued milk in combination with PEO to generate environmentally sustainable electrospun fibres [108]. Together, these studies support the dual potential of casein–polymer electrospun systems for both biomedical and eco-friendly applications, exhibiting favourable mechanical properties along with a strong antibacterial profile.

### 4.4. Other Casein-Based Delivery Systems

Recently, alternative casein-based delivery systems that go beyond conventional hydrogel and fibre formats have been explored. One approach involves nanoencapsulation, where bioactive milk-derived peptides were used in skin wound healing through cytokine production [58,116]. Applications range from microcapsule technology using glycosylated caseins [117], nanosized casein-based fibre encapsulation enhancing bioactivity [118], and even peptide caseins providing stability to patients with atopic dermatitis [58].

Lastly, an innovative system based on a nano-emulsion milk-derived cream was developed and evaluated in a thermal burn model [119]. This formulation demonstrated casein’s doubtless wound healing performance, showing anti-inflammatory properties and a skin-regenerating index (10% *w*/*w* cream showing the greatest epithelisation rate). Through diverse fabrication, the adaptability of casein for sustainable, mechanical, and therapeutic advantages is clear. Indeed, this protein constitutes a remarkable component used for developing multifunctional wound care solutions.

## 5. Functional Biological and Therapeutic Effects of Casein-Based Wound Dressings

**Figure 6:**
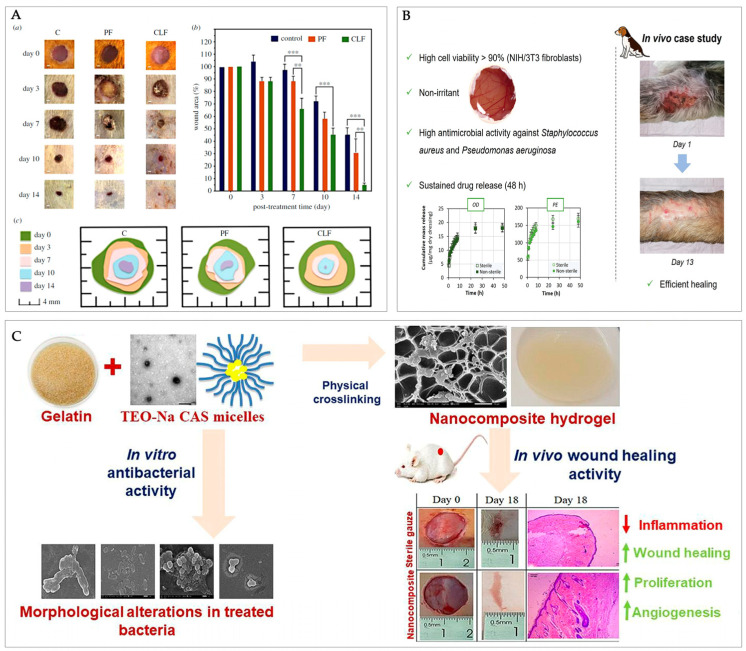
In vivo wound healing studies evidencing therapeutic efficacy of casein-based materials. (**A**) (a) Healing progress of wounds shown on days 0, 3, 7, 10, and 14 for the control (C), pure PCL fibre (PF), and casein-loaded fibre (CLF) groups (scale bar: 1 mm). (b) Comparison of wound size reduction across treatments. (c) Illustration of the healing process. Data are shown as the mean ± standard error of the mean (SEM). Significant differences are indicated as ** *p* < 0.01 and *** *p* < 0.001. [98]. (**B**) Therapeutic outcomes of a casein antiseptic-loaded hydrogel: cell viability (>90%), antimicrobial efficacy, sustained drug release, and healing findings in a canine wound case study after 13 days of treatment. Adapted from [74]. (**C**) In vivo wound healing over 18 days for a hydrogel caseinate/gelatine nanocomposite, histological evidence of reduced inflammation, and key biological findings. Adapted from [89].

Casein-based dressings have demonstrated substantial biological efficacy in wound healing applications. Their anti-inflammatory capacity is a valuable attribute for therapeutic applications. One of the most recent studies shows that casein-based dressings have demonstrated anti-inflammatory effects by reducing key pro-inflammatory cytokines such as TNF-α, TGF-β, IL-1β, NF-κB, and IL-6 [98], thereby supporting the inflammation response. Also, the immunomodulatory potential of casein-derived immunopeptides has been evidenced through their stimulation of cellular and immune functions [120]. A similar approach identified wound contraction and modulated key biomarkers, showing reduced IL-6 levels and elevated expressions of growth factor-β1 and vascular endothelial [89]. Casein-loaded fibres have shown accelerated wound closure results, nearly completing epithelial regeneration within 14 days [98] (Figure 6A). Furthermore, histological analyses have revealed casein’s capacity to support tissue formation without inducing cytotoxic effects [88,98]. In vitro cell function assays using mouse fibroblast cells [88] and along with in vivo studies involving albino male rats [89], and a dog with multiple wounds [74], consistently reported high values of cell viability (Figure 6B). This biocompatibility was further demonstrated in oil-loaded sodium caseinate (NaCAS) micelles, which supported cell proliferation while exhibiting enhanced antibacterial activity compared to free tea tree essential oil (TEO), resulting in bacterial membrane disruption and morphological distortion (Figure 6C). These nanocomposite hydrogels also showed significant in vivo wound healing efficacy, underscoring casein’s potential as a multifunctional platform for infection control and tissue regeneration [89]. The absence of significant cellular toxicity has also been confirmed in hybrid lipid-casein nanoparticle formulations [120]. This ursolic acid-loaded system exhibited high skin retention, supported cell viability in vitro, and promoted effective wound healing in vivo, further positioning casein-lipid nanocarriers as promising platforms for therapeutic delivery.

Notably, casein has contributed as an active biological protein in modulating the wound microenvironment, including humidity, temperature, and oxygen levels [121,122]. Casein-based materials can act as responsive platforms for biochemical changes when referring wound outcomes [78]. For instance, certain casein-based films can monitor proteolytic activity, where quantitative assays have correlated primary amine release with bacterial protease levels [95]. This enables hydrolysis monitoring and facilities precise wound diagnostics. Moreover, the antimicrobial potential of casein is further enhanced through the incorporation of bioactive agents [74]. For example, researchers have achieved functionalised materials with silver nanoparticles (AgNPs) [115], reporting a concentration-dependent antibacterial activity, particularly at higher silver contents [94]. Their effectiveness against *Escherichia coli*, attributed to structural sensitivity, supports their potential in antimicrobial coatings. Expanding on these developments, a recent study developed a sequentially photoactivatable hydrogel dressing using casein as a biomineralizing matrix for in situ AgNPs synthesis [123]. The system revealed robust antibacterial and photothermal performance, demonstrating sequential photoactivation potential as a novel strategy for anti-infectious wound treatment and opening new pathways for multifunctional casein-based platforms in advanced therapies.

## 6. Conclusions and Future Perspectives

Casein-based materials have demonstrated significant potential in wound management, prompting deeper investigation into their physicochemical properties. In particular, a key priority in optimising casein-based platforms is their swelling behaviour, leading to an effective exudate management [28]. Equally important are insights into their thermal sensitivity and chemical stability, including colloidal arrangements under physiological and stress conditions. Such understanding enables the design of biomaterials that respond dynamically to wound-specific factors such as moisture sensitivity [124], pH fluctuations, or enzymatic activities [88]. Since these molecular interactions contribute to the flexible structure of casein, its comprehension will guide the use of caseins as carrier material in pharmaceutical applications [28,78].

Despite promising findings, successful clinical translation requires overcoming key challenges. These include standardizing extraction and processing methods, scaling up production, and ensuring the reproducibility of casein’s bioactivity across manufacturing batches. Regulatory frameworks—such as those established by the FDA and EMA—necessitate comprehensive evaluations of biocompatibility, allergenicity, long-term toxicity, and degradation under physiological conditions. Although casein is broadly recognized as non-toxic and biocompatible, it remains a major milk allergen, especially among pediatric populations [125]. Recent studies have explored processing strategies such as enzymatic hydrolysis and thermal modification to reduce casein’s immunogenic potential [126]. Future research should aim to identify predictive biomarkers and neoepitope changes to further ensure the safety of casein-based wound care products.

While various antibacterial strategies have been employed in casein-based platforms, including silver-loaded matrices with promising outcomes, further systematic research is needed to clarify casein’s inherent antimicrobial activity. A deeper understanding of these native bioactive properties is crucial to expanding its clinical applicability. Silver nanoparticles have shown an inability to form strong physical interactions with bacterial cells, especially in multidrug-resistant bacterial-derived infections [127]. Therefore, it is suggested to conduct detailed antimicrobial long-term studies and study the implementation of novel antibacterial coatings to fully assess their suitability for wound dressing materials [94].

In summary, casein-based systems represent a promising frontier in biomedical materials. Their adaptive properties and emerging healing potential provide a strong foundation, though further research is needed to support their clinical translation and integration. Advancing this potential, their therapeutic scope now extends into specialised wound context investigations. Ongoing research has begun exploring protein fibres for surgical suturing applications [97] and casein hydrogels loaded with oxymatrine for cancer-related wound management [128]. Colostrum-derived exosomes enriched in lactoferrin and κ-casein have demonstrated enhanced anticancer effects on HepaRG cells, suggesting new possibilities for treating complicated, chronic, and oncological challenges [129]. Finally, integrating casein-based systems into clinical settings involves challenges in manufacturing logistics, in vivo model standardization, and compatibility with existing commercial wound platforms, such as hydrocolloids or foams. The incorporation of sensing technologies and customisable matrices will enable a new generation of dressing platforms, facilitating clinical adoption and a market impact in advanced wound care.
